# A pharmacovigilance study of adults on highly active antiretroviral therapy, South Africa: 2007 – 2011

**Published:** 2012-03-07

**Authors:** Nomathemba Michell Dube, Robert Summers, Khin-San Tint, Guistee Mayayise

**Affiliations:** 1 School of Health Sciences and Public Health, Faculty of Health Sciences, University of Pretoria, South Africa; 2 South African Field Epidemiology and Laboratory Training Programme, National Institute for Communicable Diseases, South Africa; 3 Medunsa National Pharmacovigilance Centre, Medunsa Campus, University of Limpopo, South Africa; 4 Department of Pharmacy, Faculty of Health Sciences, Medunsa Campus, University of Limpopo, South Africa

**Keywords:** Pharmacovigilance, human immunodeficiency virus, antiretroviral medicines, surveillance

## Abstract

**Background:**

Of the 1.6 million South African people infected with human immunodeficiency virus (HIV), approximately 970,000 (55%) have been initiated on HAART. Despite these numbers, very little has been published about the safety profile of antiretroviral (ARV) medicines in the country. This study was performed at the Medunsa National Pharmacovigilance Centre and aimed to describe the demographic characteristics of patients enrolled in the pharmacovigilance surveillance study; highly active antiretroviral therapy (HAART) initiation regimen patterns; reasons for regimen changes; and adverse effects of ARV medicines.

**Methods:**

A cohort study of HIV-infected individuals aged 15 years or older who were on ARV medicines was conducted at four sentinel sites.

**Results:**

After HAART initiation, with an average lapse of 17.8 months (range: 0 – 83.8 months), 2,815 patients were enrolled into the study. Results show that patients were observed for 1,606.2 person-years for pharmacy visits (collection of ARV medicines) and 817.1 person-years for clinical visits (consultation with the doctor). Females constituted 69.6% (1,958/2,815) of the study population. Almost all patients initiated HAART on first-line regimens (2,801/2,815). Some patients (6.7%, 190/2,815) dropped out of the study after HAART initiation. Reasons for regimen changes were not recorded for 2.5% (22/891) of the patients who changed regimens. The primary reason for regimen changes was drug-related toxicity (76.1%, 678/891), mostly evident in patients taking first-line regimens. Adverse effects experienced by patients were polyneuropathy (24.0%, 163/678); lipodystrophy (23.9%, 162/678); neuropathy (10.6%, 72/678); and suspected lactic acidosis (3.8%, 26/678).

**Conclusion:**

The majority of prescribers complied with the HAART guidelines and initiated most patients on first-line regimens. However, adverse effects are evident in patients taking first-line regimens. We recommend that the Department of Health should introduce less toxic first-line ARV regimens. Future efforts will aim to initiate patients on HAART and enrol them into the study simultaneously to determine early risk profiles of ARV medicines.

## Background

A majority of the 33 million people infected with the Human Immunodeficiency Virus (HIV) are in Sub-Saharan Africa [1]. According to South Africa's mid-year population estimates for the year 2010, approximately 5.2 million of HIV-infected people reside in South Africa [2], of which one-third need antiretroviral (ARV) medicines as their cluster of differentiation 4 (CD4) counts are below 350. Approximately 55% of South African residents who are in need of ARV medicines have been initiated on Highly Active Antiretroviral Therapy (HAART) since it was introduced into the country in the year 2003. Many others continue to seek treatment.

Although ARV medicines have been extensively used and studied in developed countries with positive outcomes being reported, such as reduction in HIV-associated morbidity and mortality [3, 4], this observation cannot be generalized to developing countries. Here, the incidence, patterns and severity of adverse reactions due to ARV medicines may differ markedly owing to local environmental and genetic influences. These influences may compromise the effectiveness of HAART programmes and lead to toxicity, intolerance, drug interactions, loss to follow-up and drug resistance amongst diverse populations such as that of South Africa. It has been found, for example, that some Acquired Immunodeficiency Syndrome (AIDS) restriction genes negatively influence HAART efficacy by delaying viral suppression and accelerating time to AIDS [5]. Therefore close monitoring of adverse drug reactions is paramount.

Pharmacovigilance (PV), an activity concerned with the detection, assessment, understanding, management and prevention of adverse reactions to medicines [6], contributes to their safe and rational use. South Africa is one of the few developing countries in Africa with an existing PV system. The Medunsa National Pharmacovigilance Centre (MNPC), functional since the year 2007, is the only PV centre in South Africa using a structured surveillance system to assess and monitor the safety profile and impact of ARV medicines in adults and adolescents.

When the roll-out of ARV medicines began in South Africa in 2003, the HAART initiation guidelines stated that all new patients needing HAART must initiate on first-line ARV regimens. First-line regimens are a combination of stavudine (d4T) or zidovudine (AZT); lamivudine (3TC); and efavirenz (EFZ) or nevirapine (NVP) [7]. Any form of resistance to these first-line regimens would require the patients to change to second-line ARV regimens. The financial and human implications of HIV drug resistance with first-line regimens are major. Second-line ARV regimens cost much more than first-line regimens [8]. Additionally, the development of drug resistance in a patient limits treatment options and increases the need for second-line regimens that maybe difficult to take [8]. Generally, second-line ARV drugs are bigger in size than first-line drugs (e.g. lopinavir/ritonavir) and therefore difficult to swallow. Some second-line drugs, such as indinavir, must be soaked in water before ingestion which makes the process of taking these ARV medicines awkward. In an attempt to broaden the options of first-line regimens, the South African government has added tenofovir, emtricitabine and others in the new South African HAART guidelines (2010).

Treatment-outcome data from a number of South African HAART programmes have been published. One such study is by Coetzee et. al. (2004) [9], published in the year 2004 when the scale-up of HAART roll-out had just begun in the South African public sector. This study aimed to promote patient adherence to ARV medicines [9]. The HAART programme has gradually evolved and improved since then. Another study conducted in South Africa by Rosen et al. (2008) [10] explored demographic and socio-economic characteristics of patients enrolled in HAART programs in private and non-governmental organization clinics around the country but excluded public health care centres.

Our study monitored patients enrolled in public sector health facilities, where the majority of HIV-infected patients in the country seek treatment. The purpose was to measure the demographic characteristics of these patients; determine their HAART initiation regimen patterns; determine reasons for regimen changes; and describe adverse effects experienced due to ARV medicines.

## Methods

### Study design, study population and study setting

This cohort study included HIV-infected patients of 15 years of age or older who were receiving ARV medicines at selected sentinel sites in the South African public health sector. HIV-infected patients not taking ARV medicines were not eligible to participate in the study. The four sentinel sites were situated in three of South Africa's provinces, namely Gauteng, Limpopo and Mpumalanga.

### Patient enrolment and data collection

Onsite coordinators based at the sentinel sites used systematic random sampling to select HIV-infected patients visiting the health facilities. Patients who agreed to participate signed an informed consent form and were enrolled into the study. PV case report forms were completed by the onsite coordinator for every new patient at enrolment and for follow-up visits. Enrolled patients’ hospital records, laboratory reports, clinician's notes and prescriptions were reviewed by the onsite coordinators. A retrospective data review of patient records was initially performed for the majority of patients to ascertain which regimen these patients initiated HAART on, as well as to obtain information on any regimens changes that had occurred prior to study enrolment of these patients. Thereafter, each time a study patient visited the health facility, they were interviewed by the onsite coordinator to identify and record any adverse effects the patient experienced while taking ARV medicines; as well to obtain information on regimen changes. All this information was recorded on the PV case report forms which were couriered to the MNPC and entered into an electronic database by data capturers based there.

### Definitions of reasons for changing or stopping regimens

During patient interviews and record reviews, the following possible reasons for changing or stopping ARV regimens were identified:

1. ARV-related toxicity, which includes adverse drug reactions (ADR) and side-effects. It is defined as the "manifestations of the adverse effects of drugs administered therapeutically or in the course of diagnostic techniques. It does not include accidental or intentional poisoning…" [11]. Such toxicity occurs at doses normally used in man and is directly linked to the presence of the drug.
2. Dose-related toxicity occurs when increased dosages of (ARV or other) medicines are linked to increased adverse effects. When the dose is reduced, so is the frequency and severity of the adverse effects. This phenomenon appears with stavudine-containing regimens, for example, and is the rationale behind the call for lower stavudine content in the relevant regimens. For the purposes of this study, ARV-related toxicity and dose-related toxicity have been distinguished as follows. ARV-related toxicity refers to an instance where one or more ARV drugs in a regimen have been changed due to adverse drug reactions. Dose-related toxicity refers to dose reductions to resolve the adverse event. A prime example is the reduction of the stavudine 40mg dose to 30mg, and even to 20mg, to limit the toxicities associated with it.
3. Treatment failure was defined as the cause for a decrease or increase in CD4 count or viral load respectively from the level it was at initiation into the study.
4. Guideline-related reasons for changes were those related to the South African HAART guidelines. For example, patients initiated on nevaripine are given 200mg doses once daily for two weeks before the dosage is increased to 200mg twice daily thereafter. The regimen/guideline related reason was meant to account for such changes that are set out by the guideline.
5. TB treatment was defined as a patient taking concurrent TB treatment and HAART. Due to possible negative consequences of combining this treatment, a decision was made to stop HAART or to change to another regimen..
6. ARV non-availability was when the regimen that the patient had initially been taking was out of stock which resulted in a patient being changed to another regimen.
7. Poor adherence was when a patient was not adhering to the prescribed regimen. This was both self-reported and obtained from pharmacy records. In the case of poor adherence, patients were usually counselled to improve adherence, after which treatment was to be re-started.
8. Patient decision was when treatment was stopped because a patient decided that s/he would discontinue HAART.
9. Pregnancy was when a patient fell pregnant whilst on a regimen containing a teratogen such as efavirenz.
10. Unknown was when a regimen had been changed but the reason for changing was not stated on the patient's hospital records.

### Data management and analysis

Data from PV case report forms were entered into a Structured Query Language database by data capturers. This data were extracted into an Excel 2007 document before being imported into Epi Info 3.5.1 (CDC) for validation. Both Epi Info 3.5.1 [12] and STATA TM version 11 (StataCorp ® LP, College Station, TX, USA) [13] were used for data analysis. Univariate analyses were performed to describe various characteristics of the study population. Categorical data were described using frequencies and proportions. Normally distributed continuous data were described using frequencies and means, while medians were used for data not normally distributed. Statistical significance was determined using the 95% confidence intervals.

### Ethical approval

The project proposal which includes the work described in this paper was submitted to and approved by the Medunsa Research and Ethics Committee at the University of Limpopo in 2006 (Project number MP119/2006).

## Results

A total of 2,835 patients was enrolled into this surveillance study since it began in January 2007 and until August 2011. Twenty of the 2,835 patients did not meet the analysis criteria because they had pharmacy records missing, therefore, they were excluded from all analyses. Only 2,815 patient data records were subsequently analysed. These patients were observed for 1,606.2 person-years for pharmacy visits (collection of ARV medicines) and 817.1 person-years for clinical visits (consultation with the doctor).

### Enrolment statistics

Of the 2,815 patients, 930 (33.0%) were enrolled at Hospital A in Gauteng province; 511 (18.2%) at Hospital B in Mpumalanga province; the lowest number of patients (171, 6.1%) at Hospital D in Gauteng province and the greatest number of patients (1,203, 42.7%) at Hospital E in Limpopo province.

### Gender and age

**Figure 1:**
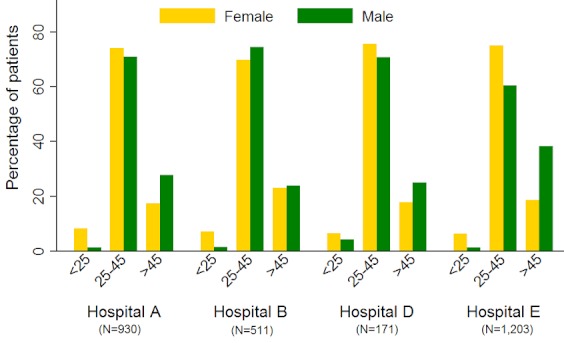
Age group at HAART initiation by gender of HIV-infected patients enrolled in the PV surveillance study, 2007 – 2011 (N=2,815)

The overall ratio of males to females was 1:2.3 (Table 1). The most frequent age of male and female patients was 42 and 39 years old respectively. Patients’ ages ranged between 18 and 79. Patients aged less than 25 years at HAART initiation comprised 5.1% of the study population (Figure 1).

**Table 1:** Univariate analysis of characteristics of HIV-infected patients on HAART and enrolled in the PV surveillance study, 2007 – 2011

Demographic Information	Males (n=856)			Females (n=1,959)		
		95% CI		95% CI
Age in years (median)	44	43 – 45	39	39 – 40
Age in years (*IQR)	38 – 51		34 – 47	
Age (in years) at HAART initiation (median)	40	40 – 41	35	35 – 36
Age (in years) at HAART initiation (IQR)	34. – 47		30– 42	
Gender (%)	29.8	28.1 – 31.5	70.2	68.5 – 72.9
**Populating group (%)**				
Black	99.6	98.9 – 99.9	99.8	99.5 – 100
Coloured	0.1	0.0 – 0.8	0.2	0.0 – 0.5
White	0.2	0.0 – 1.0	0	0.0 – 0.0
Indian	0	0.0 – 0.0	0	0.0 – 0.0

* IQR (Interquartile range)

### HAART initiation and study enrolment

The most common age group for HAART initiation amongst females was 30 – 34 years whilst that amongst males was bi-modal at 35-39 years and 40 – 44 years. In all provinces combined, 73.5% of HIV-infected patients initiated HAART at the age group 25 – 45 years old (Figure 1).

**Figure 2:**
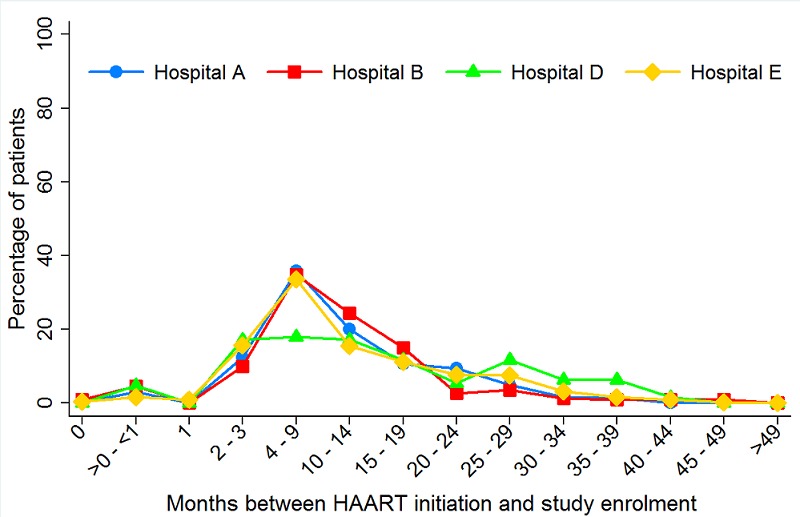
Time period between HAART initiation and PV study enrolment of HIV-infected patients, 2007 – 2011 (N=2,059)

A large number of patients (33.5%, 690/2,059) were enrolled into the PV surveillance study between four and nine months after HAART initiation (Figure 2). This calculation excluded all patients who initiated HAART before the MNPC was functional. Enrolment of patients into the study occurred at an average of 17.8 months after HAART initiation. Eight patients (0.4%) concurrently enrolled into the PV surveillance study the same day they initiated HAART. A longest interval observed between HAART initiation and study enrolment was 83.8 months. Patients who enrolled in the year 2007 had the shortest interval observed after HAART initiation.

### HAART initiation regimen patterns

The recommended first-line regimens were prescribed to 99.5% (2,801/2,815) of patients. The ARV drugs included in these regimens are shown in Table 2. Of these patients, 2.8% (80/2,815) were initiated on the recently introduced tenofovir-containing first-line regimens (regimens A1 and A2). Eleven patients initiated HAART on non-standard regimens (0.4%, 11/2,815) whilst three patients (0.1%, 3/2,815) were initiated on second-line regimens (Table 2).

**Table 2:** HAART initiation regimens prescribed to HIV-infected patients enrolled in the PV surveillance study, 2007 – 2011

HAART initiation regimens					N	%	95% CI
**First-line regimens**							
d4T*	3TC*	EFZ*		(Regimen 1a)	2,299	81.7	80.2-83.1
d4T	3TC	NVP*		(Regimen 1b)	285	10.1	9.1-11.4
AZT*	3TC	EFZ		(Regimen 1c)	118	4.2	3.5-5.0
AZT	3TC	NVP		(Regimen 1d)	19	0.7	0.4-1.1
TDF*	3TC	EFZ	or FTC*	(Regimen A1)	73	2.6	2.1-3.3
TDF	3TC	NVP		(Regimen A2)	7	0.3	0.1-0.5
*Total first-line regimens*					*2,801*	*99.5*	
							
**Second-line regimens**							
AZT	ddl*	LPV/r*		(Regimen 2)	2	0.1	0.0-0.3
TDF	3TC	LPV/r		(Regimen B1)	1	0.04	0.0-0.2
*Total second-line regimens*					*3*	*0.14*	
**Non-standard regimens**							
**Prescribers' combination**					11	0.4	0.2-0.7
**Total initiation regimens**					2,815	100	

* 3TC – lamivudine; d4T – stavudine; EFZ – efavirenz; AZT – zidovudine; NVP – nevirapine; TDF – tenofovir; FTC – emictribane; ddl – didanosine; LPV/r-lopinavir/ritonavir

### Regimen changes and stoppages

The changes and stoppages focused on in this study were those that occurred in the period between HAART initiation and the first pharmacy visit after study enrolment.

**Figure 3:**
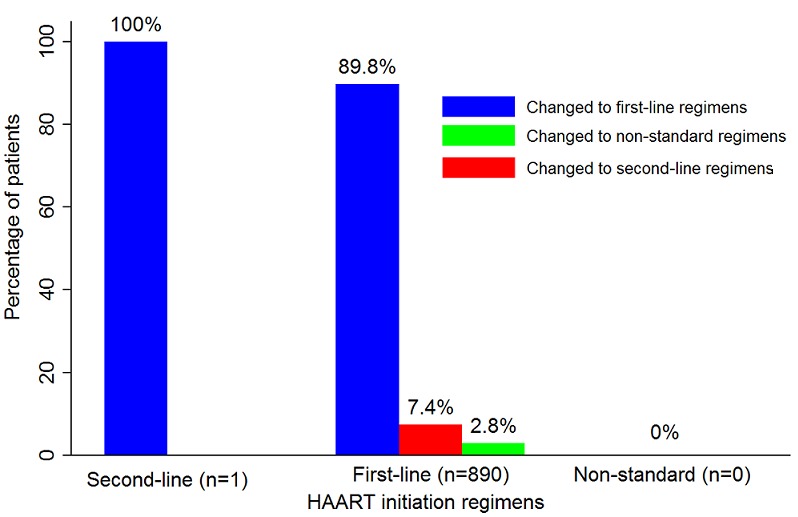
Percentage of HAART initiation regimen changes among patients enrolled in the PV study, 2007 – 2011 (N=2,815)

A total of 6.7% (190/2,815) patients did not return for their first pharmacy visit after study enrolment. These patients were classified as “drop-outs”. When grouped by initiation regimens, the numbers of drop-outs observed in descending order were as follows: regimen 1c (10.2%, 12/118; non-standard regimens (9.1%, 1/11); regimen 1a (7.4%, 170/2,299); regimen A1 (2.7%, 2/73) and regimen 1b (1.8%, 5/285). Of the patients who returned for their first pharmacy visit after study enrolment, 1.5% (40/2,625) stopped HAART temporarily. As a result of patient drop-outs and regimen stoppages 2,585 patients remained active in our study. Of these, 34.5% (891/2,585) had their initiation regimens changed. This change occurred 14.9 months, on average, after HAART initiation (95% CI: 14.2 – 15.7). Figure 3 gives a clear summary of the regimen class changes that occurred amongst patients enrolled in the study.

Amongst the patients that initiated HAART on first-line regimens, the majority of regimen changes (89.8%, 800/891) occurred within this regimen class (Figure 3). None of the patients who initiated HAART on non-standard regimens changed regimens on the first visit after study enrolment. One-third (33.3%, 1/3) of patients who initiated HAART on second-line regimens changed to first-line regimens.

### Reasons for regimen changes and stoppages

Reasons for regimen changes are outlined in Table 3. Of the patients who changed initiation regimens due to ARV-related toxicity, 76.1% (516/678) changed from regimen 1a to 1c. This number represents 22.4% of patients who commenced treatment on this regimen. Treatment failure was the second most common reason for regimen changes which was experienced mostly in patients who had initiated HAART on regimen 1a (97.2%, 70/72). To put this in perspective, 70 of the 2,299 patients who initiated HAART on regimen 1a (3.0%) changed regimens for this reason. The reason for regimen changes was not recorded in 2.5% (22/891) of patients’ files and therefore was unknown.

**Table 3:** Reasons for changing HAART initiation regimens, 2007 – 2011

Reasons for changing regimens	N	%
Drug related toxicity	678	76.1
Treatment failure	72	8.1
Pregnancy	69	7.7
Prescriber decision	19	2.1
Other reasons*	31	3.5
Not reported	22	2.5
Total (N=2,815)	891	100

* Non adherence, poor adherence, drug interaction, patient decision, ARV unavailability, guideline related

The four most common reasons given by the 40 patients (1.5%, 40/2,625) who temporarily stopped HAART were ARV-related toxicity (42.5%, 17/40), poor adherence (30.0%, 12/40), non adherence (10.0%, 4/40) and patient decision (7.5%, 3/40).

### Adverse effects of ARV medicines

Among the patients enrolled in this PV surveillance study, almost 50% (1,374/2,815) reported various medical conditions that could have been HIV and/or ARV-related. Conditions that were recorded as occurring prior to initiation of treatment were not counted as adverse effects of ARV medicines. The four most common adverse effects experienced by the 678 patients that changed ARV medicines due to ARV-related toxicity, were polyneuropathy (24.0%, 163/678), lipodystrophy (23.9%, 162/678), neuropathy (10.6%, 72/678), and suspected lactic acidosis (3.8%, 26/678). All these ARV-related toxicities were experienced by patients initiated on first-line ARV regimens 1a, 1b, 1c and A1. Approximately 96.5% (654/678) of them were experienced by patients taking regimen 1a. In relation to the number of patients enrolled in the PV study, ARV-related toxicities of each regimen in descending order were as follows: regimen 1a (28.4%, 654/2,299); regimen 1c (13.6%, 16/118); regimen A1 (4.1%, 3/73) and regimen 1b (1.8%, 5/285).

## Discussion

This is the first comprehensive report produced from the data collected at the MNPC. Very few patients enrolled into the PV surveillance study and initiated HAART simultaneously. Most patients had already been taking ARV medicines prior to study enrolment. Monitoring of adverse events associated with ARV medicines was therefore a challenge. The lowest number of enrolments was observed in Hospital D because this facility only began enrolling patients into the study in 2010. Hospital E, with the greatest number of patients enrolled is a large facility and had an onsite coordinator over the full period of the study.

At all four sentinel sites, the same demographic pattern was observed whereby females initiated HAART at an earlier age than their male counterparts. This is an indicator of gendered health seeking behavior. According to a study conducted by Skovdal et.al. (2011), males seek treatment at a later stage because they perceive themselves as physically strong and capable of withstanding disease. Additionally, they believe hospitals are places for women and children, not for men [14].

The HIV gender imbalance, where more females than males are HIV-infected, is the norm for countries with a heterosexual population like South Africa, in which older men have sexual relationships with younger women, and men have more than one sexual partner at a time [15]. This is in stark contrast to the United States and Europe populations where more males are infected with HIV than females due to the higher prevalence of homosexuality in those countries.

On examining the HIV population of the patients aged 25 years and less in this study, they seem to be under represented when compared to the same age group in a similar study in Cape Town [9]. However, this observation may support the Human Sciences Research Council (2008) data that HIV prevalence in the less than 25 year age group is declining in South Africa [16].

The number of drop-outs in our study was not as high as has been observed in similar studies in South Africa [17, 18]. A study by Dahab et al. (2008) in a South African public sector clinic revealed that 41% of patients voluntarily discontinued HAART or could not be found [19]. Reasons for patients being lost to follow-up were not assessed in our study.

The prescribing practices of health care workers attending to patients enrolled in this study were found to be in accordance to the HAART initiation guidelines (the majority of patients were initiated on first-line ARV regimens). However, the World Health Organization's (WHO) requirement that 100% of HIV-infected patients must be initiated on first-line ARV regimens was not achieved.

The four most frequently used initiation regimens in this surveillance study (regimens 1a, 1b, 1c and A1) differed from the four most frequently used regimens in a similar study in Cape Town (regimens 1a, 1c, 1d and non-standard regimens) [9]. This marked difference, where stavudine-containing regimens (regimens 1a and 1b) predominated in this surveillance study in contrast to zidovudine-containing regimens (regimens 1c and 1d) in the Cape Town study, may be due to the perception in the Cape Town cohort that treatment with stavudine is associated with peripheral neuropathy, a serious neurological complication of HIV disease. However, according to the WHO antiretroviral treatment guidelines (2007), as well as the South African HAART guidelines (2010), stavudine-associated peripheral neuropathy can be corrected by reducing the dosage of stavudine from 40mg twice daily to 30mg twice daily. Stavudine dosages as low as 20mg have been used in some patients enrolled in this PV surveillance study at Hospital A with no negative impact as yet. A study by Liddy (2009) [20] concluded that 20mg dosages of stavudine twice daily were not associated with loss of virological control and did not adversely affect CD4 count. However, due to the scarcity of available data regarding the use on the 20mg stavudine dosage, these results must be interpreted with caution.

In resource limited settings such as South Africa, older and less expensive ARV medicines such as zidovudine and stavudine are still in use despite knowledge of their toxicity profiles. This is because very few options are available in such settings. Few patients were changed from first-line to second-line and non-standard regimens in our study. The WHO recommends that the complete regimen be changed from a first-line regimen to a second-line regimen in the case of treatment failure [21]. This guideline was mostly followed in our study population where necessary.

ARV-related toxicity and treatment failure observed in our study were reported mostly in patients who initiated on regimens 1a and 1c. Although the frequency and the effects of drug-related toxicity have been assessed in clinical trials [22, 23], they have not been sufficiently evaluated in clinical settings. Persistent adverse effects cause patients to be non-adherent resulting in the HI virus becoming resistant to treatment. This process ultimately results in treatment failure of that regimen. In the majority of patients who experience regimen 1a treatment failure, the options of regimens to which they will respond well are narrowed down, which poses a major challenge to the effective ongoing treatment.

Many studies have reported the huge impact that adverse drug reactions have on health care in general and patients’ health in particular, but the actual scale of this burden cannot be accurately quantified because most cases are not reported and therefore go undetected. A study by Rajesh et al. (2010) [24] assessed the incidence of adverse drug reactions (ADRs) due to HAART in India and observed a higher incidence rate of ADRs in patients taking the zidovudine + lamivudine + nevirapine ARV drug combination than in any other ARV drug combination. This combination is equivalent to regimen 1d in our study. The results obtained in this Indian study [24] are in contrast to our study results where very few adverse effects were observed in patients taking regimen 1d. However, our results may be due to the limitation that there was a long interval between HAART initiation and study enrolment in the majority of patients. Such adverse effects may have been experienced by patients before they enrolled into our study and were therefore missed. Additionally, very few of our patients initiated on regimen 1d, which may have biased our results even further. Also, some reasons for regimen changes were not recorded in patient files, possibly leading to the underestimation of ADRs. Rajesh et al. (2010) [24] observed the lowest incidence rates of ADRs in patients taking the tenofovir + lamivudine + efavirenz ARV drug combination which is equivalent to regimen A1. Only lipodystrophy which is mostly common in patients taking stavudine-containing regimens was observed in one patient taking regimen A1 in our study. Despite this unusual observation, our results of low ADR incidence in patients taking regimen A1are consistent with those observed in the Indian study. The introduction of the new first-line regimens (regimens A1 and A2) has given health care workers more ARV options for patients to prevent drug-related side effects. It remains very important to prevent regimen changes unless absolutely necessary.

The results obtained in this study cannot be generalised to the rest of South Africa because data was only obtained from four sentinel sites located in three of South Africa's nine provinces.

## Conclusion

This PV study yields information applicable and relevant to the South African public health sector. Adverse effects of ARV medicines are evident in patients taking first-line ARV regimens. Future efforts will attempt to simultaneously initiate patients on HAART and enroll them into the study as well as to increase the number of sentinel sites to the rest of South Africa and include private health facilities. This step will help to obtain a complete profile for ADRs due to ARV medicines.

Initiating more patients on tenofovir-containing regimens may reduce the scale of ARV-related toxicity. Our recommendations therefore are that the Department of Health must look into introducing more options of less toxic first-line ARV regimens into South Africa, Health care workers in the country should be sensitized to the importance of complete reporting of reasons for regimen changes and adverse effects experienced by patients taking ARV medicines to allow correct estimation of this burden. Further research will be conducted regarding the efficacy of the 20mg dosage of stavudine.
